# Jungle Honey Enhances Immune Function and Antitumor Activity

**DOI:** 10.1093/ecam/nen086

**Published:** 2010-10-19

**Authors:** Miki Fukuda, Kengo Kobayashi, Yuriko Hirono, Mayuko Miyagawa, Takahiro Ishida, Emenike C. Ejiogu, Masaharu Sawai, Kent E. Pinkerton, Minoru Takeuchi

**Affiliations:** ^1^Department of Biotechnology, Faculty of Engineering, Kyoto Sangyo University, Kyoto, Japan; ^2^Origins Japan Co. Ltd, Nagano, Japan; ^3^Takara Shuzo Co. Ltd, Kyoto, Motoyama, Kamigamo, Kita-ku, Kyoto 603-8555, Japan; ^4^Center for Health and the Environment, University of California Davis, California, USA

## Abstract

Jungle honey (JH) is collected from timber and blossom by wild honey bees that live in the tropical forest of Nigeria. JH is used as a traditional medicine for colds, skin inflammation and burn wounds as well as general health care. However, the effects of JH on immune functions are not clearly known. Therefore, we investigated the effects of JH on immune functions and antitumor activity in mice. Female C57BL/6 mice were injected with JH (1 mg/mouse/day, seven times intra-peritoneal). After seven injections, peritoneal cells (PC) were obtained. Antitumor activity was assessed by growth of Lewis Lung Carcinoma/2 (LL/2) cells. PC numbers were increased in JH-injected mice compared to control mice. In Dot Plot analysis by FACS, a new cell population appeared in JH-injected mice. The percent of Gr-1 surface antigen and the intensity of Gr-1 antigen expression of PC were increased in JH-injected mice. The new cell population was neutrophils. JH possessed chemotactic activity for neutrophils. Tumor incidence and weight were decreased in JH-injected mice. The ratio of reactive oxygen species (ROS) producing cells was increased in JH-injected mice. The effective component in JH was fractionized by gel filtration using HPLC and had an approximate molecular weight (MW) of 261. These results suggest that neutrophils induced by JH possess potent antitumor activity mediated by ROS and the effective immune component of JH is substrate of MW 261.

## 1. Introduction

Natural products are known to have biological activity, and we have previously investigated the effect of natural products on immune function [1, 2]. Honey contains various vitamins, minerals and amino acids as well as glucose and fructose and is popular as a natural food [3–6]. There is a wide variety of honey (Manuka honey, Pasture honey, Jelly bush honey and Jungle honey, etc.), and the varieties are due to components of the flower sources. Honey is used not only as natural food but also as traditional medicine for health care, in beauty products and antiinflammatory skin care. One variety, Jungle honey, is collected from timber and blossom by wild honeybees that live in the tropical forest of Nigeria. Jungle honey is used as traditional medicine or preventive medicine to treat colds, skin inflammation and burn wounds as well as for general health care.

It is generally known that honey has antibacterial activity that has been reported to be due to its high osmolarity, acidity and presence of hydrogen peroxide and unidentified substances from floral sources [7–11]. It has been reported that flavonoid and phenol acid show antibacterial activity [12–14]. It was reported in a clinical experiment that when wound infected with gram positive and gram negative bacteria were treated with honey, infection was more quickly eradicated [15–17].

It has been reported that Manuka honey increased IL-1*β*, IL-6, and TNF-*α* production from Mono Mac6 cells or human monocytes [18, 19], and the active component was 5.8 kDa, which increased production of these cytokines via TLR4 [20]. In addition, it was reported that oral intake of honey augmented antibody productions in primary and secondary immune responses against thymus-dependent and thymus-independent antigens [21].

Honey may provide the basis for the development of novel therapeutics for patients with wounds. Therefore, the purpose of this study was to investigate the effects of Jungle honey on immune function and antitumor activity in mice.

## 2. Methods

### 2.1. Preparation of Jungle Honey

Jungle honey (JH) was a gift from Nihon origins Co. Ltd (Nagano, Japan). JH was harvested in the forest areas around the Nsukka area of Enugu state, Nigeria [22]. Generally, the bees were *Apis mellifera adonsonii*. The main plant species that the bees collected from nectar were *Pentaclethra macrophylla*, *Chrysophyllum albidum* and *Milicia excela*. Briefly, the components of JH in 100 g were 900 mg protein, 1400 mg glucon acid, 657 mg amino acid, 213 mg mineral and 3.46 mg vitamin. JH was dissolved with distilled water, freeze dried and then adjusted to 10 mg/mL with PBS(−). JH was steriled by 0.22-*μ*m filtration (Millipore, MA, USA) and then stored at 4°C before use. The endotoxin unit of JH was found to be 2.7 EU/mL using the limulus amebocyte lysate assay kit (Canbrex, MD, USA). The mice did not immunologically respond to this unit of endotoxin.

### 2.2. Mice

Female C57BL/6 mice were used at 8–10 weeks. Ten mice were used in each group. Mice were obtained from Japan SLC (Shizuoka, Japan). They were housed in transparent plastic cages with stainless wire lids in the animal facility of Kyoto Sangyo University (Kyoto, Japan). They were maintained under standard conditions, with a dark period from 8 pm to 8 am, and water and food were provided *ad libitum*. This study was approved by the committee for animals in Kyoto Sangyo University. Mice were intraperitoneally (i.p.) injected seven times with JH at a dose of 1 mg/mouse/day. Control mice received PBS(−) [Ca^2+^, Mg^2+^-free Dulbecco's phosphate buffered saline (Nissui Pharmaceutical, Tokyo, Japan)].

### 2.3. Analysis of Peritoneal Cells (PC)

Peritoneal cells were analyzed using Fluorescence Activated cell Sorter (FACS) Calibur (Becton-Dickinson, CA, USA). After seven injections of JH, PC were collected by peritoneal lavage from the mice with cold PBS. PC were pooled into plastic tubes and centrifuged at 185 g for 10 min. The pelletted cells were resuspended at 1 × 10^6^ cells/mL in FACS buffer (PBS containing 100 *μ*g/mL CaCl_2_/MgCl_2_, 0.01% sodium azide and 1% FCS). The newly appearing cell population found via Dot Plot analysis after JH exposure was sorted by FACS. The sorted cell population was suspended at 5 × 10^5^ cells/mL in R(+) (RPMI1640 containing 10% fetal calf serum, 100 U/mL penicillin, 100 *μ*g/mL streptomycin). The cell suspension (200 *μ*L of 5 × 10^5^ cells/mL) was put on a slide glass using Cyto Spin and centrifuged at 185 g for 5 min. After the slide glasses were dried in a dryer, the cells were fixed for 3 min by methanol, followed by Giemsa stain. Giemsa-stained samples were observed using a light microscope.

### 2.4. Chemotaxis Assay Using EZ-TAXIScan

Neutrophils were obtained from guinea pig peripheral blood. Blood was diluted twice with PBS. To precipitate red blood cells, peripheral blood was added in equal parts to 3.5% Dextran in saline and incubated at room temperature for 30 min. The leucocyte-rich supernatant was centrifuged at 400 g for 30 min on a Ficoll-Paque Plus (GE Healthcare, Tokyo, Japan) density gradient. The pellet was hemolyzed by hypotonic lysis. Fractionated neutrophils were centrifuged at 185 g for 10 min and resuspended at 2 × 10^6^ cells/mL in R′(+)(RPMI1640 containing 0.1% bovine serum albumin, HEPES). A chemotaxis assay for neutrophils was evaluated with EZ-TAXIScan. Time-lapse images of neutrophils during chemotaxis were obtained using EZ-TAXIScan equipped with a six channel chamber (GE Healthcare). This chamber consists of an etched silicon substrate and a flat glass plate, both of which form two compartments with a 4-*μ*m deep microchannel. Neutrophils (1 *μ*L of 2 × 10^6^ cells/mL) were put into a hole with which the device is held together with a stainless holder, and 1 *μ*L of 10^−6^ M *N*-formyl-methionyl-leucyl-phenylalanine (fMLP) or 1 mg/mL JH was put into a contra-hole. The holder assembly was filled with R′(+) and incubated for 30 min at 37°C. A charge-coupled device (CCD) camera was used to record the migration of neutrophils toward the high concentration of each sample. Cells in images were analyzed by TAXIScan Analyzer 2.

### 2.5. Antitumor Activity

Lewis Lung Carcinoma/2 (LL/2) cells were used as tumor cells. LL/2 cells were maintained in a 10-cm dish (BD Falcon, CA, USA) at 2- to 3-day intervals using MEM(+) [D-MEM (Nacalai tesque, Kyoto, Japan) containing 10% fetal calf serum, 100 U/mL penicillin and 100 *μ*g/mL streptomycin]. After 2-3 days, LL/2 were obtained with trypsin-EDTA (0.25% trypsin : 0.02% EDTA = 1 : 1, Nacalai tesque) and washed with MEM(−)(D-MEM containing 100 U/mL penicillin and 100 *μ*g/mL streptomycin). Hemocytometer and trypan blue dye exclusion testing were used to determine LL/2 total number and viability. Mice injected with JH or PBS(−) were inoculated intraperitoneally with LL/2 (4 × 10^5^ cells/0.2 mL/mouse). After 4 weeks, antitumor activity was evaluated by tumor incidence and weight. Tumor tissues were fixed in 10% neutral buffered formalin fixative and paraffin embedded. Sections (4 *μ*m) were stained with H&E.

### 2.6. Production of Reactive Oxygen Species

PC (1 × 10^5^ cells/100 *μ*L) were incubated with Hydroethidine (HE, Polysciences, PA, USA, final concentration 10 *μ*M) or 2′,7′-Dichlorofluorescin diacetate (DCFH-DA, Sigma, MO, USA, final concentration 20 mM). After a shaking incubation at 37°C for 30 min, the cells were washed twice and resuspended in 200 *μ*L of PBS(+) (Dulbecco's phosphate buffered saline) and then analyzed using FACS.

### 2.7. Fractionation of Jungle Honey

JH (100 mg/mL) was fractionized from Fr. 1 to Fr. 5 by gel filtration using a Shodex OHpak SB-802 HQ column and HPLC (LC-20AD, RID-10A, SPD-20A, CB-20A, Simazu, Japan). Elution was carried out with PBS(−) at a flow rate of 1 mL/min for 30 min. Standards curves were traced using polyethylene glycols, which had MW of 3930, 1020, and 106 (Polymer Laboratories, Germany), and LCsolution GPC (Shimau), under the same conditions in HPLC. The MW of JH was estimated using the polyethylene glycol standard curves. Each fraction of JH was freeze dried and then adjusted to a concentration of 10 mg/mL with PBS(−).

### 2.8. Expression of IL-1*β* mRNA

Aliquots of obtained PC (1 × 10^5^ cells/100 *μ*L/well) were cultured with or without JH fractions (final concentration 500 *μ*g/mL) in 96-well flat bottom culture plates (Becton-Dickinson, MA, USA) at 37°C for 24 h in 5% CO_2_. After 24 h, total RNA was isolated by acid guanidinium thiocyanate-phenol-chloroform assay. Total RNA was transcribed to cDNA with MLV reverse transcriptase (Invitrogen, CA, USA). Oligonucleotide primers were used from published cDNA sequences of IL-1*β* (250 bp) and *β*-actin (268 bp) (house-keeping gene). PCR was performed for 30 cycles using the following primer pairs: *β*-actin sense (5′-GCATTGTTACCAACTGGGAC-3′) and *β*-actin antisense (5′-TCTCCGGAGTCCATCACAAT-3′); IL-1*β* sense (5′-AGCTACCTGTGTCTTTCCCG-3′) and IL-1*β* antisense (5′-GTCGTTGCTTGGTTCTCCTT-3′). The amplification profile consisted of denaturation at 94°C for 30 s, primer annealing at 56°C for 30 s and extension at 72°C for 30 s. PCR products were visualized using ethidium bromide after 8% polyacrylamide gel electrophoresis. Data on the expression in IL-1*β* mRNA were quantified by Scion image.

### 2.9. Statistical Analysis

All values are expressed as mean ± SE. Comparisons between control and JH-injected mice were made with the Student's *t*-test. Any *P*-values < .05 were considered statistically significant.

## 3. Results

### 3.1. Increases of the Number of PC by Jungle Honey

The number of PC was significantly (*P* < .001) increased in JH-injected mice (5.13 ± 0.28 × 10^6^ cells/mouse) compared to control mice (1.17 ± 0.11 × 10^6^ cells/mouse).

### 3.2. Induction of New Cell Populations of PC by Jungle Honey

**Figure 1:**
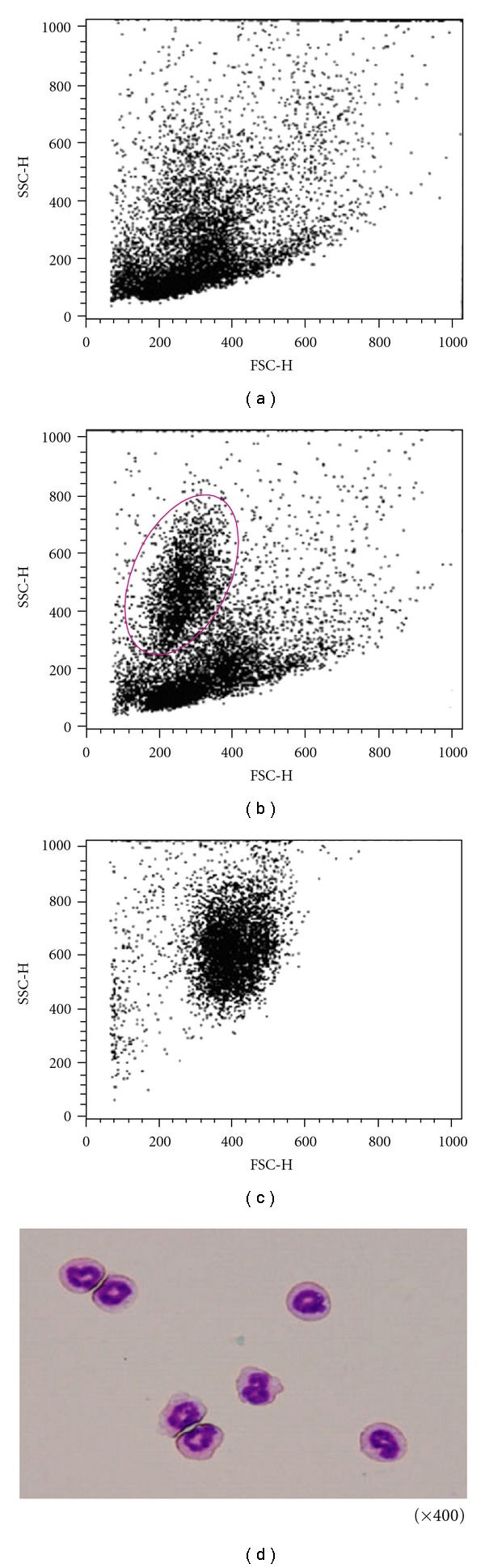
Induction of new cell populations of PC by Jungle honey. (a) Control. (b) Jungle honey. (c) Isolated cell population. (d) Isolated cell population.

New cell populations in JH-injected mice were found at FSC 120–400, SSC 200–800 by Dot Plot analysis of FACS (Figure 1(b)) compared to control mice (Figure 1(a)). An isolated, new cell population was found, as is shown in Figure 1(c) and the isolated cells were found to be neutrophils (Figure 1(d)) by Giemsa stain. The new cell population in JH-injected mice was observed by a light microscope and was identified as neutrophils by their morphology (Figure 1(d)).

### 3.3. Enhancement of Chemotaxis for Neutrophil by Jungle Honey

**Figure 2:**
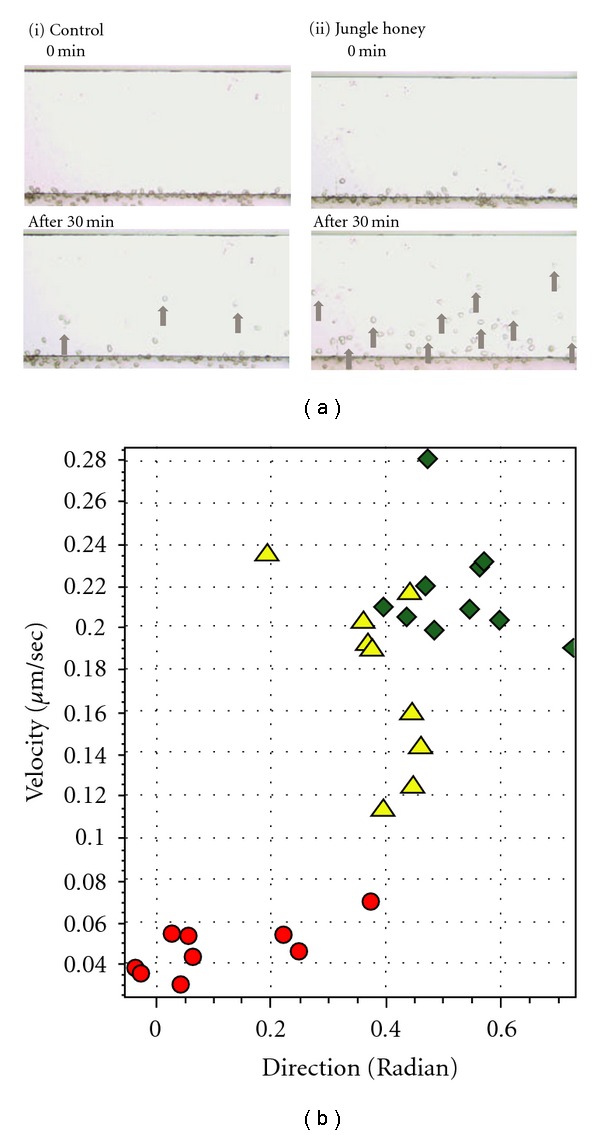
Enhancement of chemotactic activity for neutrophils by Jungle honey. (a) Image of neutrophil chemotaxis. (b) Dot plots of velocity and direction of neutrophils. Up arrow: Migrated cells, filled circle: control, filled diamond: fMLP (10^−6^ M), filled triangle: JH (1 mg/mL).

Forty neutrophils migrated in 30 min in the JH-treated group compared to 13 neutrophils in the non-treated group (Figure 2(a)-i, ii). The velocity of the migrating neutrophils was 0.17 ± 0.01 *μ*m/s in the JH-treated group and 0.04 ± 0.01 *μ*m/s in the non-treated group (Figure 2(b)). The radian of neutrophils was 0.39 ± 0.04 rad in JH-treated group and 0.09 ± 0.03 rad in the non-treated group (Figure 2(b)). JH resulted in significantly (*P* < .001) increased numbers, velocity, and radiation of migrated cells. Therefore, JH showed chemotactic activity for neutrophils.

### 3.4. Inhibition of LL/2 Tumor Growth by Jungle Honey

**Figure 3:**
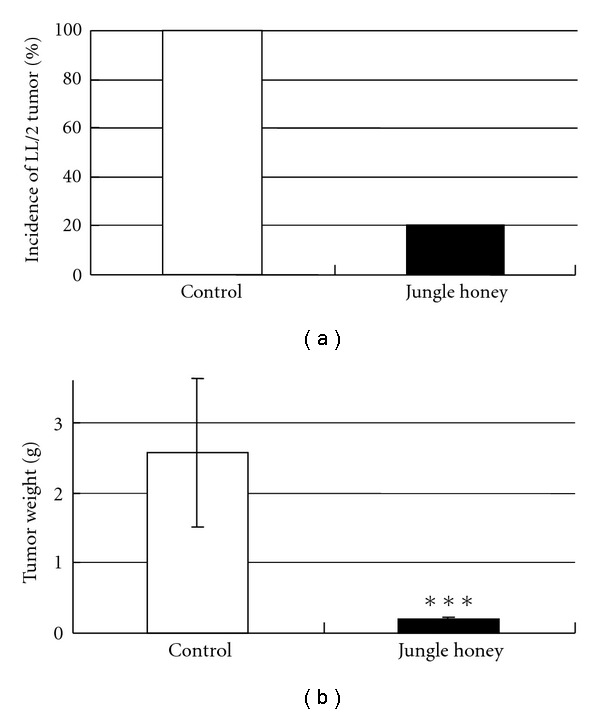
Inhibition of the incidence of LL/2 tumor (a) Tumor weight (a) by Jungle honey.

**Figure 4:**
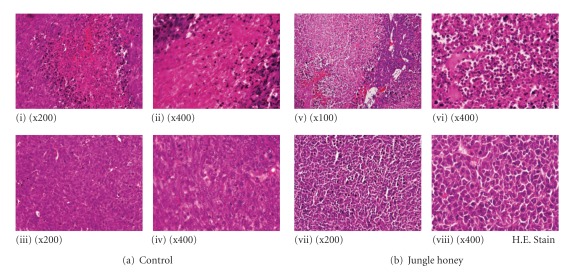
Histological findings of LL/2 tumor by Jungle honey. (a) Control. (b) Jungle honey.

The incidence of LL/2 tumors was 20% in JH-injected mice and 100% in control mice (Figure 3(a)). The mean tumor weight was 0.02 ± 0.02 g in JH-injected mice and 2.57 ± 1.05 g in control mice (Figure 3(b)). These results reveal that JH inhibited tumor incidence and growth (Figure 4). In histological findings of control tumor tissue, necrotic areas were recognized (Figure 4(a)-i, iii), but there were few infiltrations of neutrophils (Figure 4(a)-ii, iv). In JH injected-tumor tissue, massive necrotic areas (Figure 4(b)-v) and infiltration by many neutrophils were observed (Figure 4(b)-vi). Hemorrhagic necrotic areas and a disassociation between tumor cells were found (Figure 4(b)-vii, viii).

### 3.5. Increases of ROS Production in PC by Jungle Honey

**Figure 5:**
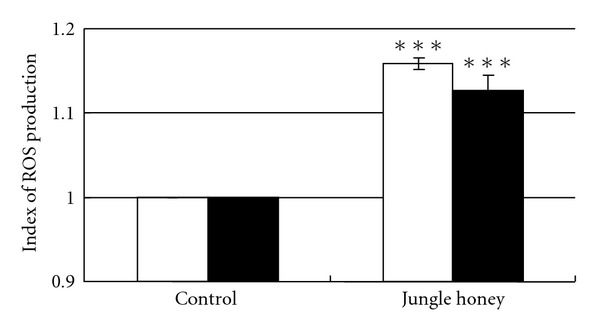
Increases of ROS production in PC by Jungle honey. Open square: O_2_^−^, filled square: H_2_O_2_.

The ratio of control was 1.0. The ratio of O_2_^−^ was 1.16 and the ratio of H_2_O_2_ was 1.13 in the JH treated group. The ratio of O_2_^−^ or H_2_O_2_ producing cells were significantly (*P* < .001) increased in JH-injected mice compared with control mice (Figure 5). Therefore, Reactive Oxygen Species (ROS) may be associated with antitumor activity.

### 3.6. Enhancement of IL-1*β* mRNA Expression in PC by Jungle Honey Fractions

**Figure 6:**
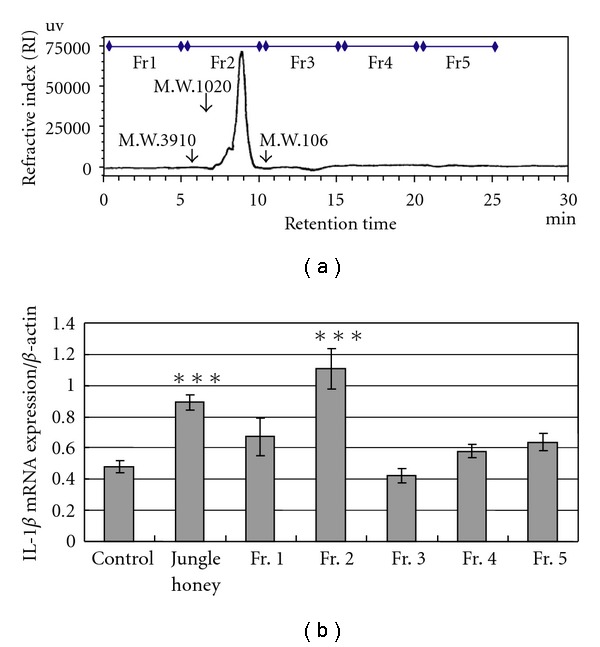
Enhancement of IL-1*β* mRNA expression in PC by Jungle honey fractions. (a) Fraction by gel filtration. (b) IL-1*β* mRNA expression.

IL-1*β* mRNA expressions by JH and each fraction from Fr. 1 to Fr. 5 were 0.94 ± 0.06, 0.67 ± 0.12, 1.11 ± 0.13, 0.42 ± 0.05, 0.58 ± 0.04 and 0.64 ± 0.05, respectively (Figure 6(a)). IL-1*β* mRNA expressions were significantly (*P* < .001) increased by JH or Fr. 2 (Figure 6(b)).

## 4. Discussion

Jungle honey (JH) is collected from timber and blossom by wild honeybees that live in the tropical forest of Nigeria, where JH is used as traditional or preventive medicine for colds, skin inflammation and burn wounds as well as general health care. Therefore, we expected that JH would have potential biological, especially immune, activity. Until now, the effect of JH on immunomodulatory activity has been relatively unknown. Therefore, we investigated the effects of JH on immune function and antitumor activity in mice.

We found that the number of peritoneal cells (PC) was increased *∼*4-fold in JH-injected mice compared with control mice. This result suggests that JH induces cell migration. Although the effects of other types of honey on PC numbers are not yet reported, mice treated with other natural products (i.e., hydroalcoholic extract from *Chenopodium ambrosioides* or aqueous extract from *Orbignya phalerata Mart*) have been shown to have increased PC as well [23, 24].

To characterize the new cell population found after JH treatment, we investigated surface antigens by FACS. In Dot Plot analysis, a new cell population appeared in the region of FSC 120–400 and SSC 200–800 in JH-injected mice. The percent of Gr-1 surface antigen and the intensity of Gr-1 antigen expression of PC were increased in JH-injected mice (data not shown). Moreover, the new cell population was found to be neutrophils based on morphology. Although the effects of honey on Dot Plot and cell surface antigen of PC were not reported, it was shown that the number of neutrophils was increased in treated mice with propolis [25]. This result agrees with our present report.

Our results showed that JH may have chemotactic activity for neutrophils. Therefore, we investigated the chemotactic activity of JH for neutrophils. The velocity and direction of migration were increased by JH compared with the control treatment. These results suggest that JH possesses chemotactic activity for neutrophils. Although there are no reports concerning chemotactic activity of honey, polysaccharide from *Ganoderma lucidum* have chemotactic activity for neutrophils in the Boyden chamber assay [26].

Because it was demonstrated that the number of PC and migration of neutrophils were increased by JH, we investigated antitumor activity by immune cells. LL/2 tumor cells were used as syngeneic tumor cells, which have low-tumor antigen and inhibit the immune system as well as human cancer [27]. The incidence and the mean weight of LL/2 tumors were decreased in JH-injected mice compared to control mice. These results suggest that JH has a preventive effect on tumor growth. Tumor weight was found decreased by royal jelly, propolis or polyphenol in propolis [28–30]. The tumor tissue was infiltrated by many neutrophils at massive necrotic areas in JH-injected mice. Therefore, it was suggested that the neutrophils were involved with inhibition of tumor growth.

To investigate the mechanism of antitumor activity by JH, we examined ROS, a well-known antitumor factor. The ratio of ROS produced by cells was increased in JH-injected mice. Although the effect of honey on cellular ROS production has hereto been unreported, one study reported that H_2_O_2_ production was increased in PC treated with extracts of *G. lucidum* or *P. cornucopiae* [31]. This result agrees with our present study. It was reported that ROS produced by activated neutrophils has tumor cytotoxic properties as well as preventive action against infection [32–35]. Since infiltration of many neutrophils was observed at necrotic areas in JH injected-tumor tissue, there is a possibility that antitumor activity by JH is due to production of ROS by infiltrated neutrophils into tumor tissue.

JH was fractionized from Fr. 1 to Fr. 5 by gel filtration using HPLC to identify the effective component in JH, and IL-1*β* mRNA expressions in PC were assayed using each fraction. IL-1*β* augments immune response, functions and migration capability of neutrophils. JH and Fr. 2 augmented the expression of IL-1*β* mRNA in PC. Therefore, the effective component was Fr. 2 in JH, and the effective component in Fr. 2 was estimated to have a MW of 261. In contrast, it was reported that the effective component of honey or royal jelly was 55 kDa (Apalbumin-1), and the effective component of Manuka honey was 5.8 kDa; these two components also were found to increase TNF-*α* production [19, 36–38]. Therefore, the active component of honey or royal jelly and Manuka honey differs from the active component of JH.

Our results suggested that JH-induced neutrophils to the peritoneal cavity, and the neutrophils were activated by IL-1*β*, which was produced by JH stimulation. Then ROS from activated neutrophils was associated with antitumor activity. In addition, the effective component in JH was found to have a MW of 261.

## Funding

Grant-in-Aid for Scientific Research (C) in Japan Society for the Promotion of Science (Grant no. 20500606).
